# Supplementary material for: Sézary Syndrome in West Sweden: Exploring Epidemiology, Clinical Features, and Treatment Patterns in a Registry-Based Retrospective Analysis
Source: Cancers (Basel). 2024 May 21;16(11):1948. doi: 10.3390/cancers16111948 (PMC11171299; doi:10.3390/cancers16111948)
Supplement: Supplementary file 1 [file cancers-16-01948-s001.zip › cancers-3015464-supplementary.pdf]

Supplementary Materials: The following supporting information can be downloaded at: www.mdpi.com/xxx/s1, Table S1: Occupations in 17 patients with Sézary syndrome; Table S2: Summary of comorbidities in 17 patients with Sézary syndrome; Figure S1: Clinical characteristics of Sézary syndrome patients. a: onychodystrophy; b: keratoderma; c: leonine facies; d: erythroderma.

Table S1. Occupations in 17 patients with Sézary syndrome

Patient number	
1	Project manager at a refrigeration company, work on farms with pesticides
2	House painter, electromechanical technician
3	Truck driver
4	Nursing assistant, seamstress
5	Firefighter, craftsman
6	Not applicable
7	Seaman, truck driver, warehouse manager
8	Service occupations, working in a school
9	Childcare worker
10	IT consultant
11	Accounting consultant
12	Sales assistant, farmer
13	Bus driver
14	Not applicable
15	Not applicable
16	Seaman
17	Electrician

Table S2. Summary of comorbidities in 17 patients with Sézary syndrome.

Comorbidities	n	Comorbidities	n
Actinic keratoses	1	Herpes simplex 1	1
Appendicitis	1	Herpes simplex 2	2
Asperger's syndrome	1	Hyperlipidemia	7
Atrial flutter	1	Hyperparathyroidism	2
Atrial fibrillation	3	Hypertension	14
Benign prostatic hyperplasia	4	Hypertrophic cardiomyopathy	1
Blepharitis	1	Hypothyroidism	2
Cataracts	4	Impaired hearing	2
Cholecystitis	1	Inguinal hernia	3
Chronic kidney disease	1	Ischemic stroke	3
Congestive heart failure	4	Myocardial infarction	2
COPD	2	Myoma	1
Deep vein thrombosis	3	Nonalcoholic fatty liver disease	1
Depression	2	Osteoarthritis	4
Diabetes mellitus	5	Peroneus pares	1

Diverticulitis	1	Pityrosporum folliculitis	1
Endocarditis	1	Pneumonia	1
Esophagitis	1	Pneumothorax	1
Facial palsy (peripheral)	1	Pulmonary embolism	3
Gallbladder polyps	1	Renal incidentaloma	1
Glaucoma	2	Rosacea	1
Gout	1	Seborrheic dermatitis	1
Graft versus host disease	1	Sepsis (Staphylococcus aureus)	1
Hand eczema	1	Sleep apnea	2
Hepatitis B	1	Tinea corporis	1
Hepatitis C	1	Urinary incontinence	1
Herniated disc	1		

COPD: Chronic obstructive pulmonary disease

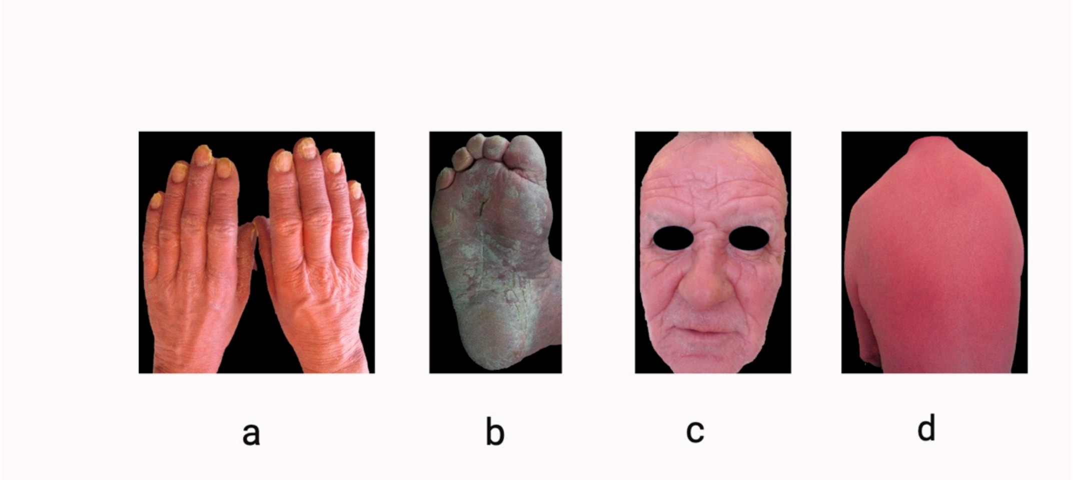

Figure S1. Clinical characteristics of Sézary syndrome patients. a: onychodystrophy; b: keratoderma; c: leonine facies; d: erythroderma.
